# Supplementary material for: SPOT: A machine learning model that predicts specific substrates for transport proteins
Source: PLoS Biol. 2024 Sep 26;22(9):e3002807. doi: 10.1371/journal.pbio.3002807 (PMC11426516; doi:10.1371/journal.pbio.3002807)
Supplement: S1 Text — (DOCX) [file pbio.3002807.s003.docx]

**Computing task-specific protein representations**

ESM-1b protein representations are computed by taking the element-wise mean of all updated 1280-dimensional amino acid representations resulting from the ESM-1b model that was trained in a self-supervised manner. However, simply taking the element-wise mean of all amino acid representations can result in information loss and does not take into account the prediction task for which the representations are to be used, which can lead to suboptimal performance [1]. We attempted to overcome these problems by creating task-specific transport protein representations optimized for predicting transporter-substrate pairs. Analogous to a previous study [2], we slightly modified the architecture of the ESM-1b model by adding an additional token to represent the whole transporter to capture information relevant to the downstream prediction task. This whole-transporter representation was mapped to the 1280-dimensional embedding space of the amino acid embeddings and was updated in the same way as the regular ESM-1b amino acid representations. After a predefined number of update steps, the transporter representation was concatenated with the ECFP-vector for a potential substrate molecule. The combined vector was used as the input for a fully connected neural network (FCNN), which was then trained end-to-end to predict whether the molecule is a substrate for the transporter. This approach facilitates the construction of an optimized, task-specific vector representing the complete transport protein sequence. After training, we used the network to extract the 1280-dimensional task-specific representations for all transporters in our dataset. We call these representations ESM-1b_ts_ vectors.

**References**

1. Detlefsen NS, Hauberg S, Boomsma W. Learning meaningful representations of

protein sequences. Nat. Commun. 2022 Apr 08;13(1):1914

2. Kroll A, Ranjan S, Engqvist MKM, Lercher MJ. A general model to predict

small molecule substrates of enzymes based on machine and deep learning. Nat.

Commun. 2023;14(1):2787.
