# Supplementary figures and images for: SPOT: A machine learning model that predicts specific substrates for transport proteins
Source: PLoS Biol. 2024 Sep 26;22(9):e3002807. doi: 10.1371/journal.pbio.3002807 (PMC11426516; doi:10.1371/journal.pbio.3002807)

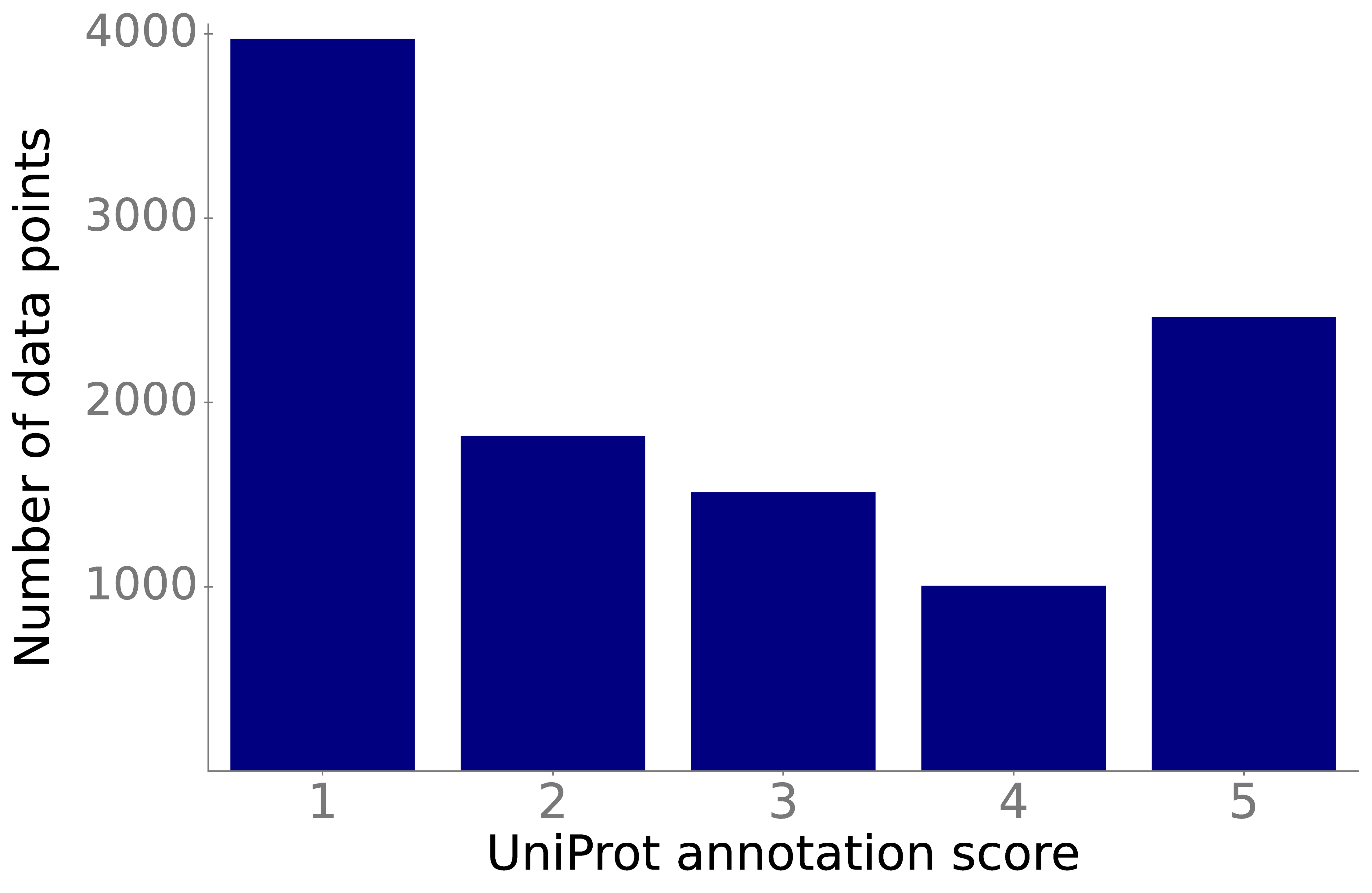

Supplement: S1 Fig — UniProt IDs extracted from the TCDB are grouped by their annotation scores in the UniProt database. The annotation scores range from 1 to 5, where higher values indicate better evidence for the protein annotation. Direct experimental evidence corresponds to a score of 5. The data underlying the graph shown in this figure can be found in S2 Data. (TIF) [file pbio.3002807.s006.tif]

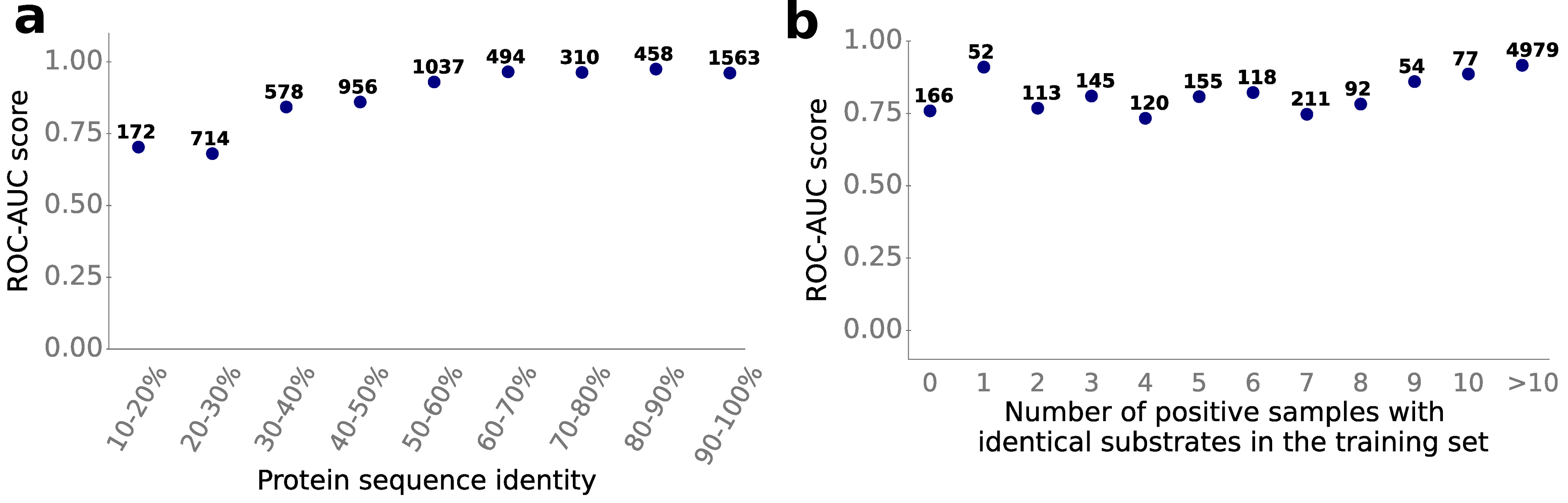

Supplement: S2 Fig — (a) We divided the test set into different subsets according to the maximal pairwise protein sequence similarities compared to all proteins in the training set. We calculated the ROC-AUC score for each subset. (b) We grouped small molecules by how often they occur as substrates among all positive data points in the training set. We calculated the ROC-AUC score for each group. The data underlying the graphs shown in this figure can be found in S2 Data. (TIF) [file pbio.3002807.s007.tif]

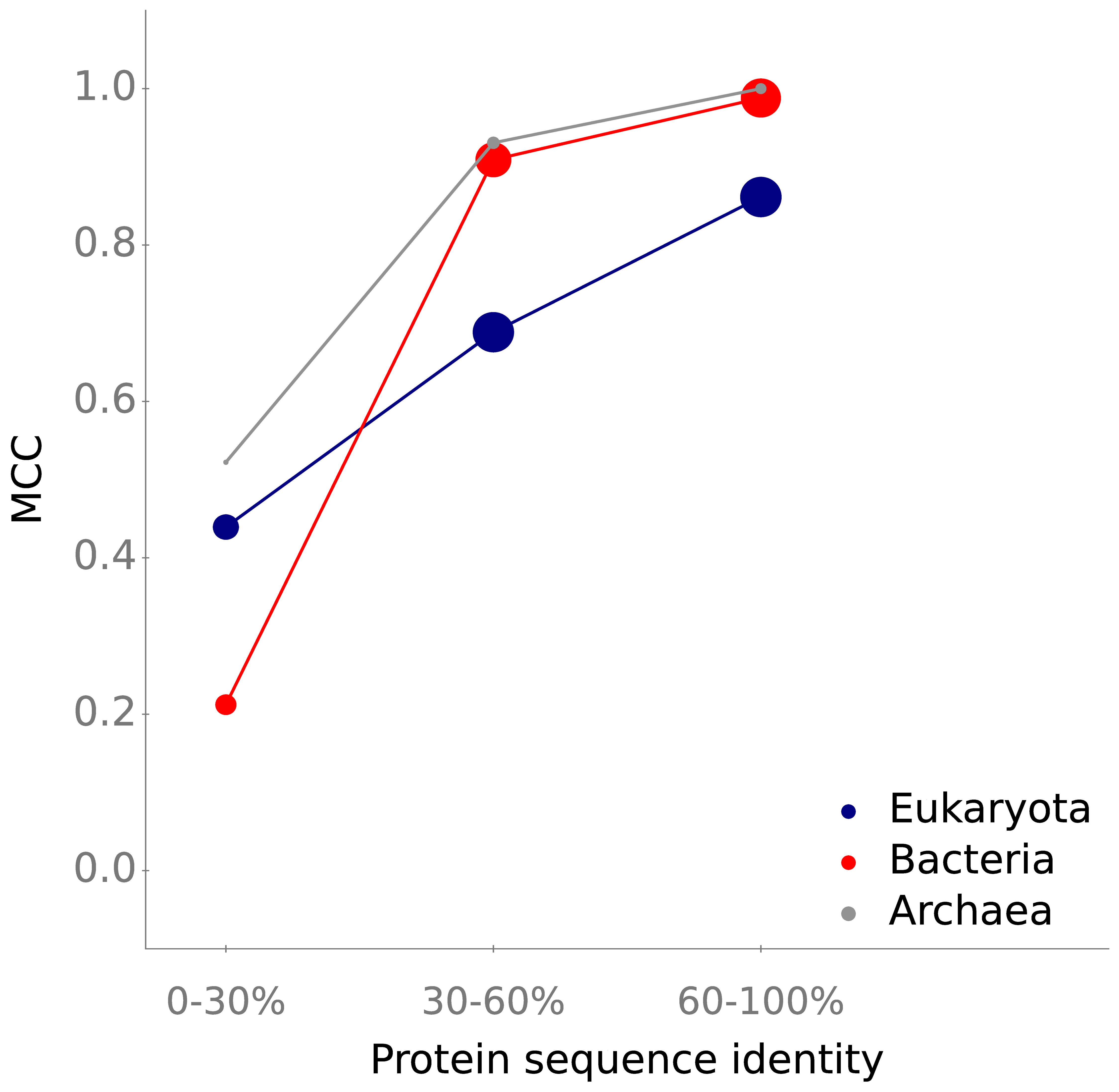

Supplement: S3 Fig — We divided the test set into 3 classes based on the domain of the source organism of the transport protein: Eukaryota, Bacteria, and Archaea. We further divided each class into subsets according to the maximum sequence identity compared to the training proteins. The plot shows the Matthew’s correlation coefficient (MCC). The areas of the circles are proportional to the number of data points in each subset. The data underlying the graph shown in this figure can be found in S2 Data. (TIF) [file pbio.3002807.s008.tif]

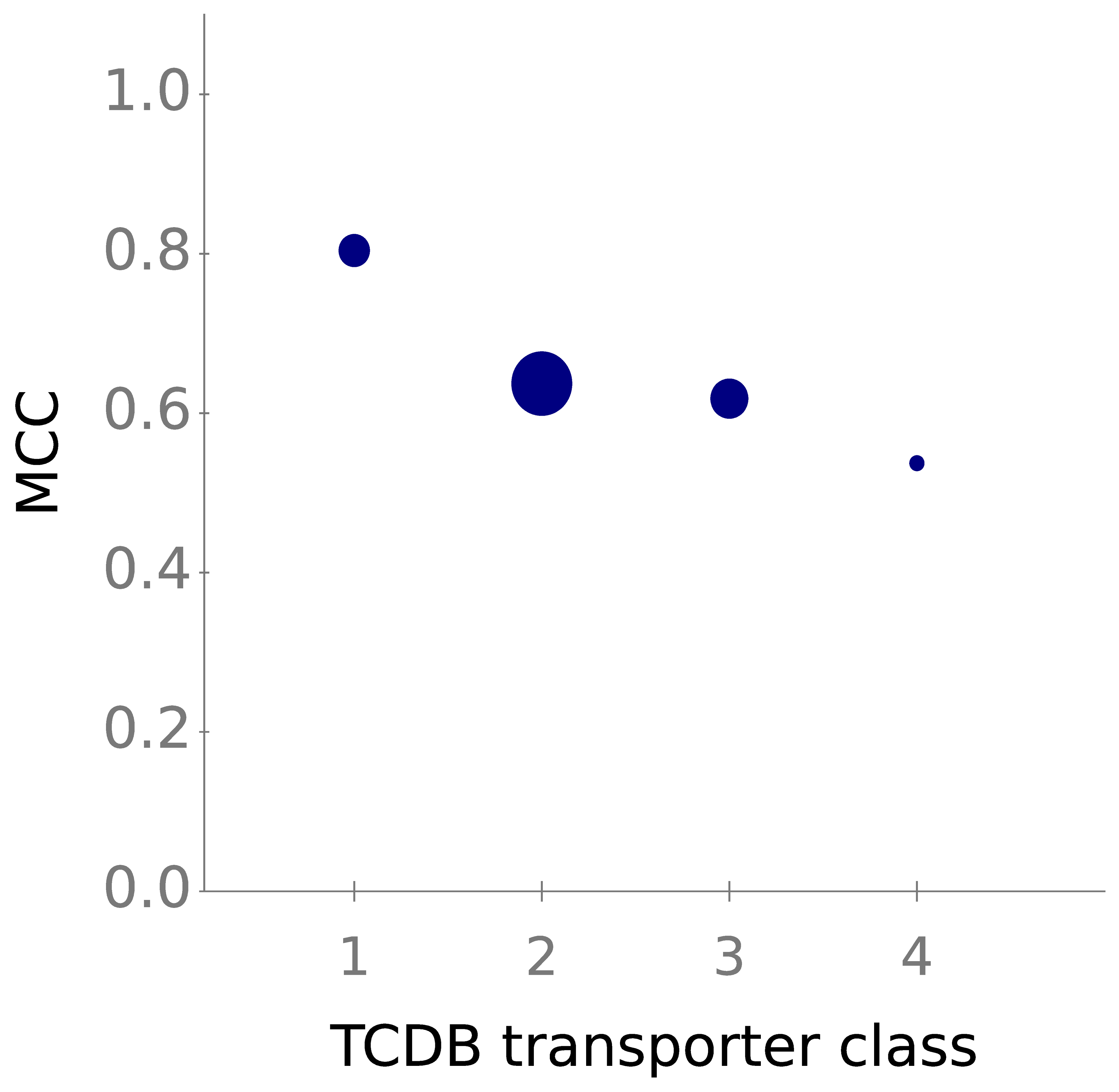

Supplement: S4 Fig — We partitioned the test set into 4 distinct classes based on transport mechanisms annotated in TCDB: channels/pores (class 1); electrochemical potential-driven transporters (class 2); primary active transporters (class 3); and group translocators (class 4). The plot shows the Matthew’s correlation coefficient (MCCs) for data points from each class. The areas of the circles are proportional to the number of test data points in each subset. The data underlying the graph shown in this figure can be found in S2 Data. (TIF) [file pbio.3002807.s009.tif]

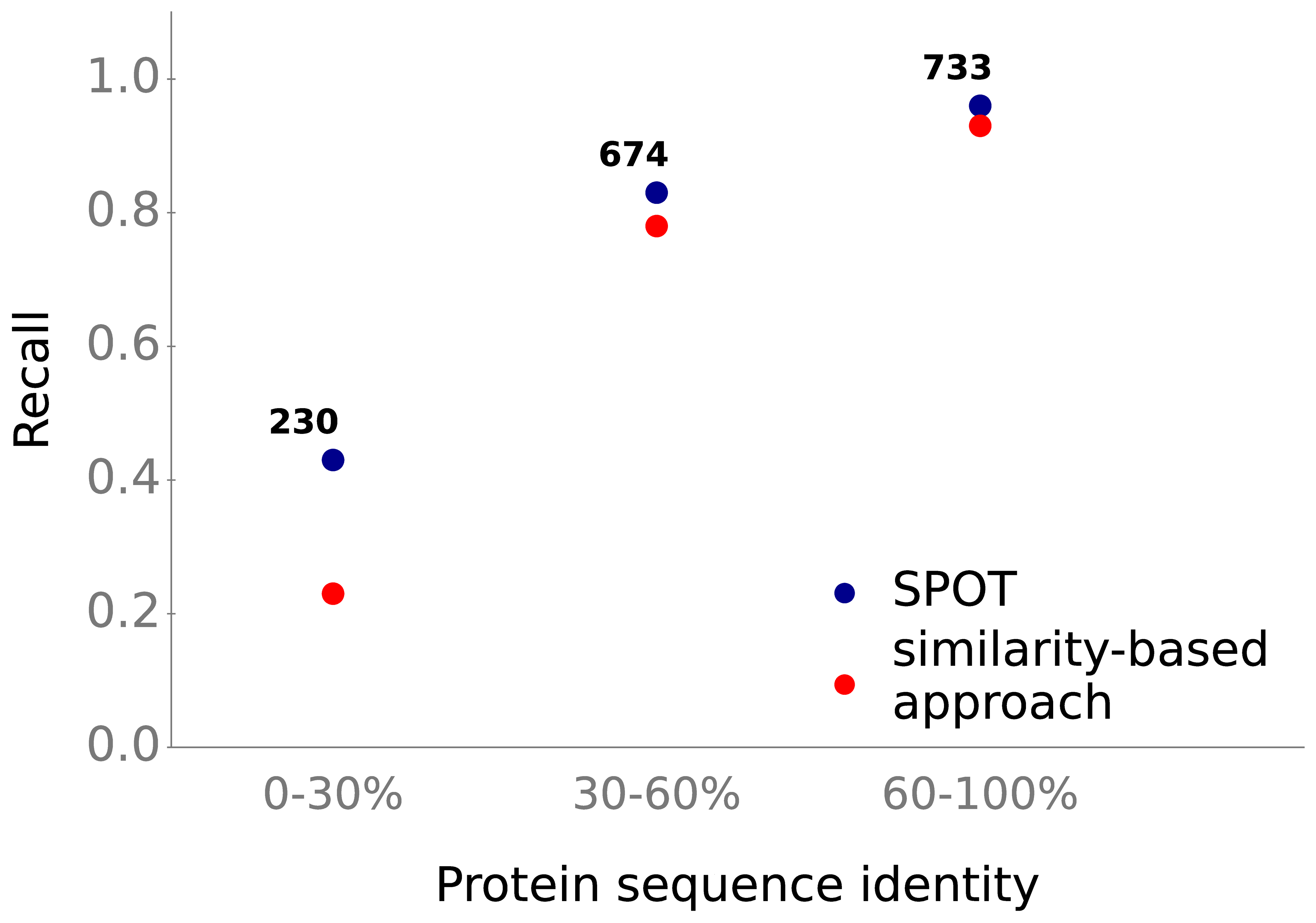

Supplement: S5 Fig — We divided the test set into subsets based on the maximum pairwise protein sequence similarities compared to all proteins in the training set. For each subset, we computed the recall of SPOT and a simpler, similarity-based method that considered the substrates of the most similar protein from the training set. The data underlying the graph shown in this figure can be found in S2 Data. (TIF) [file pbio.3002807.s010.tif]

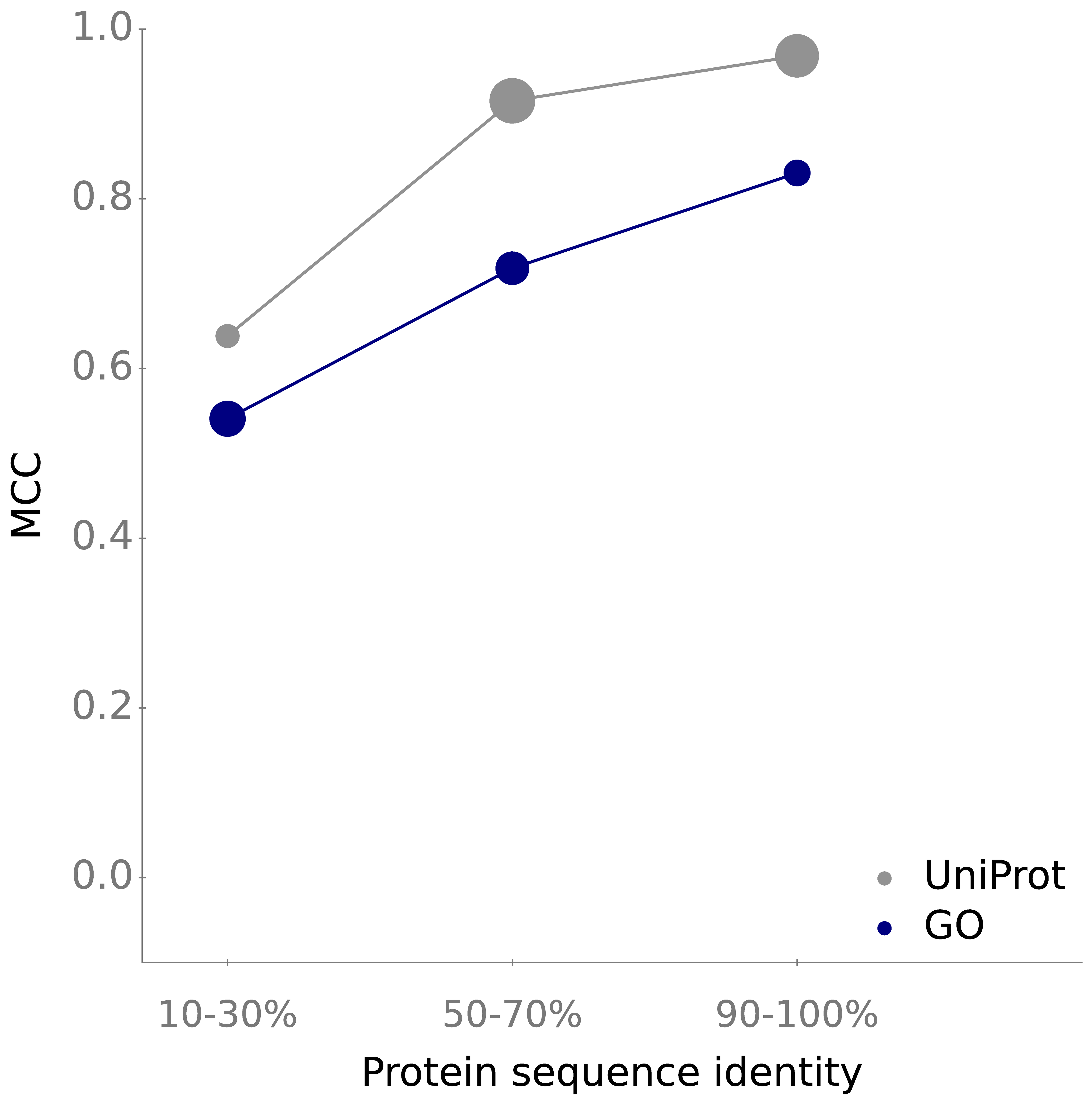

Supplement: S6 Fig — We partitioned the test set into 2 subsets based on their origin—UniProt or GO. The plot shows the Matthew’s correlation coefficient (MCC) for different levels of maximal sequence identity compared to training proteins. The areas of the circles are proportional to the number of test data points in each subset. The data underlying the graph shown in this figure can be found in S2 Data. (TIF) [file pbio.3002807.s011.tif]
